# Evaluation of modified atmosphere packaging system developed through breathable technology to extend postharvest life of fresh muscadine berries

**DOI:** 10.1002/fsn3.4037

**Published:** 2024-03-18

**Authors:** Uzman Khalil, Ishtiaq A. Rajwana, Kashif Razzaq, Shehbaz Singh, Ali Sarkhosh, Jeffrey K. Brecht

**Affiliations:** ^1^ Department of Horticulture MNS‐University of Agriculture Multan Pakistan; ^2^ Horticultural Sciences Department University of Florida Gainesville Florida USA; ^3^ Curation Foods Inc. Santa Maria California USA

**Keywords:** antioxidant, berry firmness, decay, fruit quality, storage, *Vitis rotundifolia*

## Abstract

Muscadine grapes (*Vitis rotundifolia* Michx.) are delicate in nature with short shelf life. Postharvest technologies like modified atmosphere packaging (MAP) with reduced oxygen (O_2_) and elevated carbon dioxide (CO_2_) could increase the postharvest storage life with better quality. In the current experiment, physical and biochemical quality attributes of black and bronze cultivars of muscadine grapes ('Supreme' and 'Granny Val', respectively) were evaluated in active MAP. Fruit were packed in plastic trays, sealed with impermeable film, and CO_2_ was introduced into the package. The MAP was created by a rigid microperforated plastic patch coated with a proprietary semipermeable resin, which was applied over a hole in the tray; packages with the same size hole without a patch were the control. Fruit were stored at 4°C for 42 days (6 weeks). MAP resulted in significantly lower decay incidence and better retention of fruit firmness for up to 28 days of storage in both cultivars as well as reducing color changes in 'Supreme' fruit. Although MAP did not affect the biochemical quality of muscadine grapes, total antioxidants increased initially and then decreased during storage, irrespective of packaging treatments. A significant linear increase in total phenolic content was also found during storage, regardless of treatments applied. Overall, the results of the current study demonstrate that MAP can be an affective technology to increase storage duration of muscadines with better retention of physical quality, without affecting the biochemical attributes.

## Graphical abstract

Modified atmosphere packaging (MAP) developed through breathable patch can be an affective technology to increase storage duration of muscadine grapes with better retention of physical quality, without affecting the biochemical attributes. MAP resulted in significantly lower decay incidence and better retention of fruit firmness as well as reducing color changes in 'Supreme' cultivar. Although it did not affect the biochemical quality of muscadine grapes, total antioxidants increased initially and then decreased during storage, irrespective of packaging treatments.

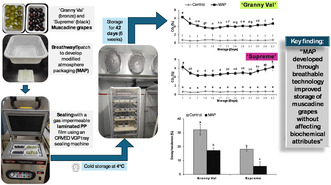

## 1 INTRODUCTION

Muscadines (*Vitis rotundifolia* Michx.) are rich in health‐promoting compounds like polyphenols. The thick skin of muscadine grape berries is rich in pigments and polyphenols, including anthocyanins, tannins, quercetin, flavan‐3‐ols, gallic acid, ellagic acid, ellagic acid glycosides, ellagitannins, myricetin, and kaempferol (Andersen et al., 2020). Muscadines are native to the southeastern USA and are successfully grown there because of the plant's hardy nature and resistance to the endemic pests and diseases of the region. Muscadine plants have natural resistance against Pierce's disease, which is a major limiting factor for growing common grapes (*Vitis vinifera* L.) in Florida (Andersen et al., 2020). Unlike common grapes that are harvested in bunches, muscadines do not form bunches and thus the ripe berries are individually harvested (Khalil, Rajwana, Razzaq, Brecht, et al., 2023).

Muscadine grapes have a short shelf life and about 50% of the berries can suffer from decay within 7–10 days at ambient temperature (Walker et al., 2001). Notable reduction in decay incidence of muscadines was achieved when the berries were stored at 0 or 4.5°C in comparison to 20°C (Takeda et al., 1983). At refrigerated or low‐temperature storage, muscadines can be stored up to 2–3 weeks (Perkins‐Veazie et al., 2012), but they are susceptible to postharvest softening, shriveling, weight loss, and decay when stored longer (Walker et al., 2001).

Being nonclimacteric, muscadines must be allowed to ripen completely before harvest and they have a very low respiration rate compared to climacteric fruit. Although the quality of the fruit deteriorates during storage due to physiological breakdown, the decay caused by microorganisms is the major cause of fruit losses during storage. Common grapes can be stored for about 6 months in cold storage along with fumigation with sulfur dioxide (SO_2_) or by using SO_2_ pads in packaging to reduce decay (Thompson, 2015). However, SO_2_ fumigation is not recommended for muscadine because of its bleaching effect on the berries and off‐flavor development (Smit et al., 1971; Takeda et al., 1983). Moreover, usage of SO_2_ is restricted in many countries because of health concerns, especially for people allergic to sulfites. Therefore, alternative methods of decay control need to be explored to extend muscadine grape's storage life.

Controlled atmosphere (CA) storage involves altering gas concentrations, especially reducing the oxygen (O_2_) and elevating the carbon dioxide (CO_2_), in addition to low temperature, which can increase the storage duration of perishable fruit and vegetables (Thompson, 2018). Metabolic activity of fruit is reduced under CA storage conditions that increase shelf life. We previously reported that muscadines were quite tolerant of high CO_2_ concentrations (up to 30%) during postharvest storage and that CA storage maintained better fruit quality and reduced decay incidence compared to air storage for up to 42 days at 4°C (Shahkoomahally et al., 2021). Smittle (1990) reported that muscadines can be stored for 6 weeks in a CA of 5% O_2_ plus 15% CO_2_ at 1.1–2.2°C.

The use of modified atmosphere packaging (MAP) for extending storage life of fruit and vegetables is a well‐established technology. Recent studies have shown promising effects of MAP on the storage life and quality of various perishable fruits and vegetables like raspberry, mulberry, figs, persimmon, banana, bell pepper, and jujube (Huynh et al., 2023; Islam et al., 2022; Kızıldeniz et al., 2023; Li et al., 2023; Liamnimitr et al., 2018; Lwin et al., 2022; Wang et al., 2023). Using MAP involves designing a package to control gas exchange with the outside environment so that the product respiration and the package permeability interact to achieve a beneficial steady‐state atmosphere within the package that, like CA, consists of reduced O_2_ and elevated CO_2_. The reduced O_2_ can result in attenuated product respiration, which slows metabolism by restricting the supply of adenosine triphosphate (ATP), as well as potentially reducing biosynthesis of ethylene, which requires O_2_; elevated CO_2_ primarily benefits produce storage by reduction decay due to its fungistatic effect (Brecht et al., 2020). There are two types of MAP systems, active and passive (Zagory & Kader, 1988). In active MAP, the desired atmosphere is quickly established by flushing the package with a gas mixture during sealing, while in passive MAP the steady‐state atmosphere is slowly established by the interaction between product respiration and package permeability; the same interaction maintains the atmosphere previously established in active MAP (Lange, 2000).

MAP has been reported to retain better quality during storage of many common grape cultivars (Artés‐Hernández et al., 2003, 2004; Martínez‐Romero et al., 2003; Yamashita et al., 2000). For muscadine, there was remarkably less decay when the berries were held in vented plastic clamshell packages that were wrapped in polyethylene bags, although the resulting modified atmosphere (MA) was not reported (Walker et al., 2001). A problem with MAP is that the response of product respiration to changes in temperature is much greater than the response of the plastic film permeability to the same changes (Beaudry et al., 1992). The BreatheWay® MAP system (Curation Foods, Inc., Santa Maria, CA, USA) addresses this issue by coating a rigid microperforated plastic patch with a semipermeable resin, the permeability of which dramatically differs above and below a designed phase transition temperature (Clarke, 2011). We recently reported on the use of BreatheWay® MAP to improve the shelf life of pink tomatoes (Batziakas et al., 2022).

Therefore, the current study was conducted to investigate the impact of an active MAP on muscadine quality during storage including the biochemical composition of the fruit. In the current experiment, we used BreatheWay® MAP technology to achieve the desired MA, and its impact on physicochemical quality of muscadine grapes was evaluated. The novelty of this study is that the MAP was maintained through a self‐adjusting breathable technology, and its impact was evaluated on both the physical and biochemical quality of muscadine grapes, which are entirely different from common vinifera grapes.

## 2 MATERIALS AND METHODS

### 2.1 Fruit supply

Individual berries of two muscadine grape cultivars, bronze‐colored 'Granny Val' and black 'Supreme', were harvested, based on their color development, from a commercial muscadine vineyard in Wray, Georgia, USA. The berries were transported in insulated coolers to the postharvest laboratory at the Horticultural Sciences Department, University of Florida in Gainesville, FL (approx. 250 km or 3 h), where they were held overnight at 4°C. The following morning, damaged or diseased berries were removed, and only healthy and uniform berries were selected for further use.

### 2.2 Fruit MAP

Muscadine berries were packed in 650‐ml gas‐impermeable polypropylene plastic trays (Model 1/8 Gastronorm H45, Orved S.p.A., Venice, Italy) with dimensions of 14.5 × 9.5 × 4.3 cm (*L* × *W* × *D*) that were heat‐sealed over the tray opening with a gas‐impermeable 85‐μm thick laminated polypropylene film (ORVED OPA PP) using an ORVED VGP tray sealing machine. Berries were placed in a single layer inside the package and the fruit weight inside each package ranged from 181 to 227 g. For the active MAP treatment, a CO_2_ gas supply was connected to the sealing machine to flush during tray sealing and establish a targeted 6% CO_2_ inside the packages. We had previously determined that 6% CO_2_ is sufficient to maximize decay control in muscadines, while low O_2_ is relatively ineffective (Shahkoomahally et al., 2021). The CO_2_ flushing procedure ensured immediate establishment of the desired gas composition inside the package so that the fruit quality would not be affected by delay in developing the MAP due to the low respiration rate of muscadines (i.e., this was an active MAP system). On one side of the plastic tray, a hole of 4 mm diameter was made and based on preliminary tests, approximately half of the area of the hole was covered with plastic tape. For the MAP, a microporous patch with polymer coating (BreatheWay®, Curation Foods) was then pasted over the hole (Figure S1) to maintain the desired modified atmosphere (MA) inside the packages, which can adapt to temperature as well as respiration changes within the package. This technology helps in maintaining the desired gas mixture inside the package. Identical packages with the same hole size without a microporous patch were used as the control. This procedure was employed based on preliminary tests to restrict air movement in the packages to limit water loss without modifying the package atmosphere with regard to the respiratory gases.

### 2.3 Fruit storage

After packing, the berries were stored in a cold storage room at 4 ± 0.1°C and 95% relative humidity (RH) to represent the typical temperature of many commercial storage facilities and of household refrigerators of consumers. Trays were kept on wire shelf units with a plastic curtain covering the top and sides of each shelf unit to avoid direct contact with air from the room fans. The experiment was laid out in such a way that each of the three replicates per treatment from both cultivars was evaluated after every 2 weeks in storage for 6 weeks.

### 2.4 Concentrations of carbon dioxide and oxygen

Concentrations of CO_2_ and O_2_ inside three packages per treatment and cultivar were recorded twice per week during storage using a PBI Dansensor CheckMate 9000 Gas Analyzer (PBI, Ringsted, Denmark). A needle was inserted through the tray wall to the inside of the package to take gas readings and after taking each reading, the hole in the packaging was sealed with plastic tape.

### 2.5 Evaluation of physical parameters

Weight loss on a fresh weight basis was evaluated by recording the weight of trays of berries at the beginning of the experiment and at the time of final evaluation. Decay incidence was calculated from the decayed berries out of the total number of berries in each tray and expressed as a percentage.

To record color and firmness, 10 berries from each of three replicates at each evaluation time were selected randomly. Color was recorded from two opposite sides of each berry using a chromameter (Konica‐Minolta, CR‐400, Osaka, Japan) and expressed as L*, a*, and b* values, with the a* and b* values being converted to hue angle and chroma. Firmness was recorded using a FirmTech 2 (Bioworks Inc., Wamego, KS, USA) instrument and 10 berries were subjected to the compression test to calculate the firmness from each replicate, which is expressed by the instrument as the resistance per unit distance of compression (g per mm^−1^).

### 2.6 Evaluation of biochemical attributes

For evaluation of various biochemical attributes, 10 berries from each of three replicates at each evaluation time were blended after removal of seeds (leaving the edible portion of peel and flesh) and centrifuged at 19,319 × g_n_ for 20 min at 4°C. The resulting supernatant was used to determine total soluble solids (TSS) and titratable acidity (TA). The TSS was recorded using an automatic temperature‐compensated refractometer (Reichert, Inc., Reichert r2i300, NY, USA). To record TA, 3.0 mL of fruit juice was titrated against 0.1 N NaOH to an endpoint of pH 8.2 using an automatic titrator (Metrohm 814 USB Sample Processor, Herisau, Switzerland). The methods for TSS and TA are as described by Shahkoomahally et al. (2021). The TA was expressed as the percentage of acid in the fruit juice based on the molecular weight of tartaric acid, the predominant organic acid in muscadine.

### 2.7 Total antioxidants, total phenolic contents, and anthocyanins

Total antioxidants from the juice were determined using the ferric reduction antioxidant power (FRAP) assay method described by Benzie and Strain (1996). Trolox (6‐hydroxy‐2,5,7,8‐tetramethylchroman‐2‐carboxylic acid) was used as the standard solution and the absorbance from each plate having standards and sample was recorded at 595 nm using a microplate reader (Epoch 2; BioTek Instruments, Inc., Winooski, VT, USA). Total antioxidants were expressed as mg kg^−1^ on a fresh weight basis for Trolox equivalents.

Total phenolic content (TPC) was determined using the Folin–Ciocalteu reagent method and the absorbance was recorded at 765 nm, using the method described by Singleton (1966) with some modifications. Gallic acid (GA) was used to make a standard curve and TPC was expressed as GA equivalents on a fresh weight basis and expressed as mg kg^−1^.

Anthocyanins were determined using the pH differential method (Giusti & Wrolstad, 2001). Sodium acetate (0.40 M, pH 4.5) and potassium chloride (0.025 M, pH 1.0) buffers were used to determine anthocyanins. The absorbance of samples was recorded at 510 and 700 nm using a BioTek microplate reader and anthocyanins were calculated using the formula described by Shahkoomahally et al. (2021).

### 2.8 Statistical analysis

Statistical analysis was performed as analysis of variance with two factors (MAP treatments × storage days) using MINITAB® 16 statistical software (Minitab Inc., PA, USA). The data were treated for multiple comparisons with least significant differences test (Fisher's LSD) at *p* ≤ .05 level of significance.

## 3 RESULTS

### 3.1 Concentrations of carbon dioxide and oxygen

**FIGURE 1:**
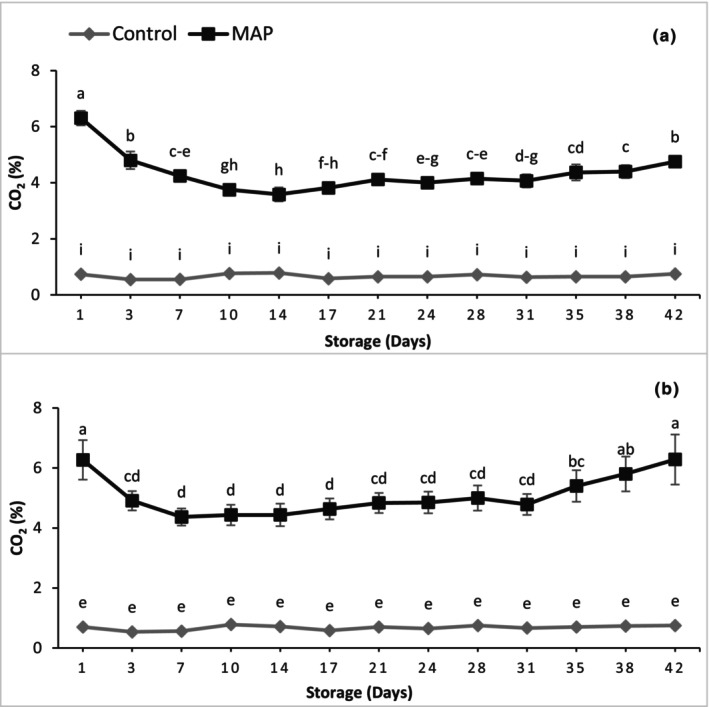
Effect of MAP on the package headspace CO_2_ levels for (a) 'Granny Val' and (b) 'Supreme' muscadine grape berries over 42‐day storage at 4°C. Data presented here are the mean values of three packages and different letters indicate significant differences between treatments and sampling times (*p* ≤ .05).

Muscadines were packed in sealed trays with BreathWay® patches to develop MAP. In MAP, the CO_2_ concentration decreased from 6.29% initially to a minimum of 4.01%, averaged over the two cultivars, then started increasing gradually, whereas the control trays did not show any significant change in CO_2_ during the entire storage duration (Figure 1). For 'Granny Val', a 1.76‐fold decrease in CO_2_ concentration was recorded after 14 days of storage. Later, CO_2_ started slowly increasing from 14 to 42 days of storage, reaching 4.75%. Like 'Granny Val', 'Supreme' also followed a similar decreasing trend of CO_2_ concentration in the beginning, but the minimum CO_2_ concentration for 'Supreme' (4.37%) was recorded after 7 days of storage in MAP. This minimum CO_2_ concentration was 1.44‐fold less as compared to Day 1 and started increasing afterward; on the last day of storage, it was 1.44‐fold higher than Week 1 reaching 6.28% of CO_2_. Regardless of storage days, MAP resulted in 6.49‐ and 7.48‐fold higher CO_2_ than the control for 'Granny Val' and 'Supreme', respectively.

**FIGURE 2:**
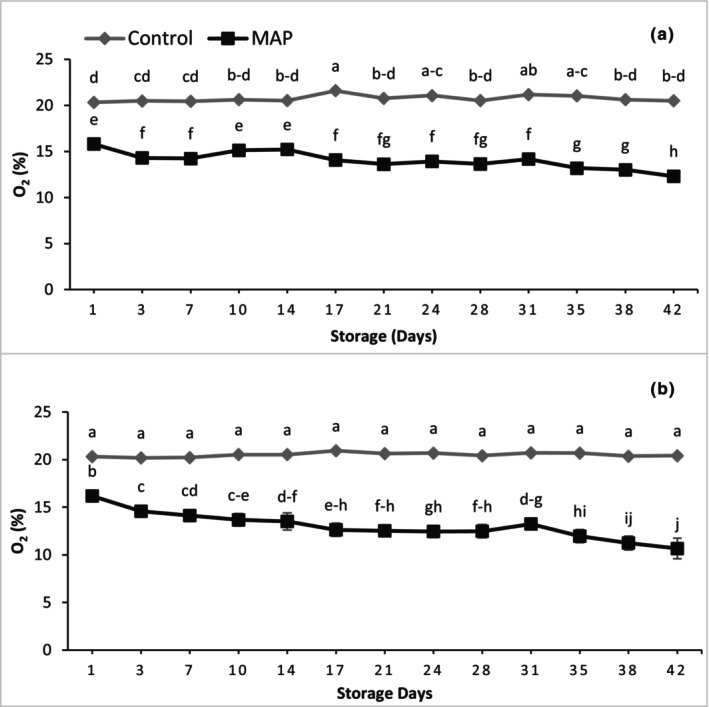
Effect of MAP on the package headspace O_2_ levels for (a) 'Granny Val' and (b) 'Supreme' muscadine grape berries over 42‐day storage at 4°C. Data presented here are the mean values and different letters indicate significant differences between treatments and sampling times (*p* ≤ .05).

The concentrations of O_2_ for both cultivars did not change significantly during the entire storage duration in the control trays without MAP (Figure 2). Overall, the O_2_ level decreased during storage in MAP for both cultivars. For 'Granny Val', the O_2_ level decreased significantly for 3 days after sealing the tray, from 15.8 to 14.2%, which became stable over the first 28 days as the apparent decline was not statistically significant. After 28 days of storage, the O_2_ level started decreasing significantly up to the last day of storage, reaching 12.3% O_2_. For 'Supreme', a linear decreasing trend of O_2_ was recorded during the entire storage duration, but the rate of decreasing O_2_ was higher from 28 to 42 days declining from 12.5 to 10.7%. MAP resulted in significantly lower O_2_ concentrations than the control, 1.48‐fold and 1.58‐fold less in 'Granny Val' and 'Supreme', respectively.

### 3.2 Physical parameters

**FIGURE 3:**
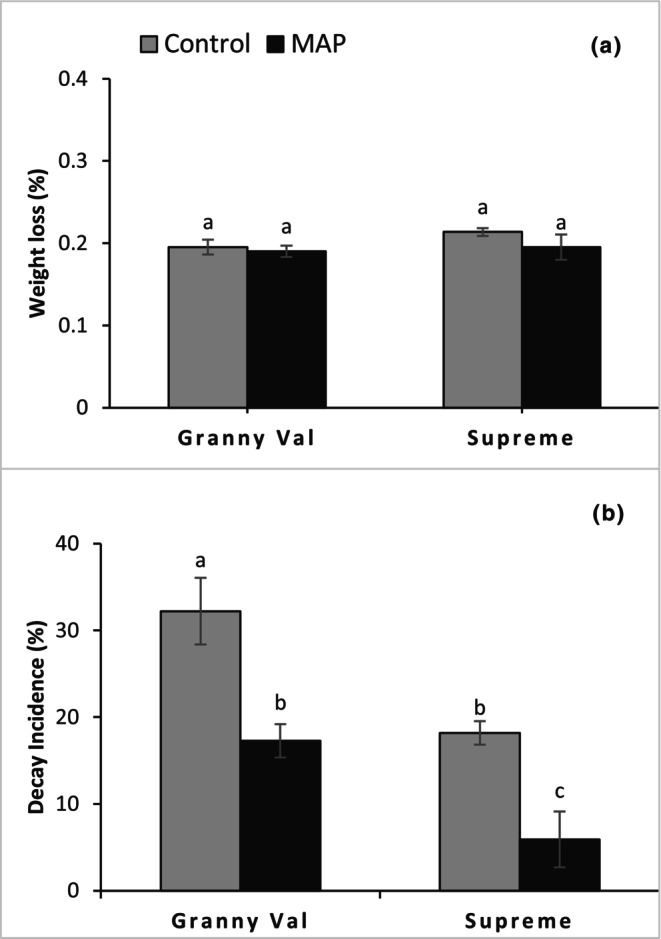
Effect of MAP on the weight loss (a) and decay incidence (b) for 'Granny Val' and 'Supreme' muscadine grape berries after 42 days of storage at 4°C. Data presented here are the mean values and different letters within column clusters indicate significant differences between treatments (*p* ≤ .05).

Berry weight loss was not affected significantly in response to MAP (Figure 3a) but reduction in decay incidence was highly significant (Figure 3b). Lower decay incidence was recorded in 'Supreme' stored in either MAP or control (5.9% vs. 18.2%) than that in 'Granny Val' (17.3% vs. 32.3%) after 42 days. Comparing the cultivars, decay incidence was 2.05‐fold less in 'Supreme' (12.1%) than in 'Granny Val' (24.8%), irrespective of the treatments.

MAP reduced the color changes in both grape cultivars during storage, but the effect was much more pronounced in 'Supreme' than in 'Granny Val' (Table 1; Figures S1 and S2). For the bronze‐colored 'Granny Val' grapes, the L* decreased linearly over the storage time and differed slightly between the two treatments, with MAP retaining higher L* up to the last day of storage, at which time the L* value in MAP was 2.0 units higher than the control. The a* value of 'Granny Val' berries increased linearly during storage, but there was no significant effect of MAP until the last day of storage, at which time the a* value in MAP was lower than in the control. At the end of storage, the a* value of 'Granny Val' had increased by about 65%, but remained in the negative (green) range. The b* value and chroma changed very little during storage of 'Granny Val', declining only slightly, but more so in the control than in MAP. The hue of 'Granny Val' berries was not affected significantly by MAP, but there was a significant linear decrease of about 4 degrees during storage.

**TABLE 1:** Effect of MAP on color parameters of 'Granny Val' and 'Supreme' muscadine grape berries over 42‐day storage at 4°C.

Treatment	Storage (days)	Granny Val					Supreme				
		L*	a*	b*	Chroma	Hue (°)	L*	a*	b*	Chroma	Hue (°)
Control	0	46.8a	−3.5c	15.7a	16.1a	102.4a	26.5a	4.6a	1.7a	4.9a	21.7a
	14	43.8bc	−2.8b	14.8a	15.1a	100.4b	29.1b	3.2a	4.2a	6.8a	36.7a
	28	43.1c	−2.4b	14.8a	15.1a	98.9b‐d	32.1bc	2.2a	6.8a	8.9a	49.6a
	42	41.0d	−1.8a	13.7a	13.9a	97.6d	31.3c	2.3a	6.0a	8.2a	48.1a
MAP	0	46.8a	−3.5c	15.7a	16.1a	102.4a	26.5a	4.6a	1.7a	4.9a	21.7a
	14	44.7a	−2.8b	15.5a	15.8a	100.3b	27.2a	5.0a	2.8a	6.4a	27.3a
	28	44.0a	−2.5b	15.6a	15.9a	99.4bc	28.0a	3.8a	3.4a	6.2a	33.1a
	42	42.9a	−2.4b	15.1a	15.3a	98.8cd	27.3a	3.6a	3.2a	5.9a	31.6a

*Note*: Data presented here are the mean values and different letters within column indicate significant differences between treatments (*p* ≤0.05).

For 'Supreme', a linear increase in values of L*, b*, chroma, and hue was recorded up to 28 days, which decreased slightly afterward, irrespective of treatment. The L* remained lower in MAP as compared with the control and, on the last day of storage, the difference was 4.0 units. The a* value decreased linearly during the storage irrespective of the applied treatments, but MAP fruit retained 37.6% higher a* value until the last day of storage. The values of b*, chroma, and hue were retained at lower values in MAP with a difference of 3.4‐, 1.4‐, and 1.5‐fold, respectively, at the end of storage.

**FIGURE 4:**
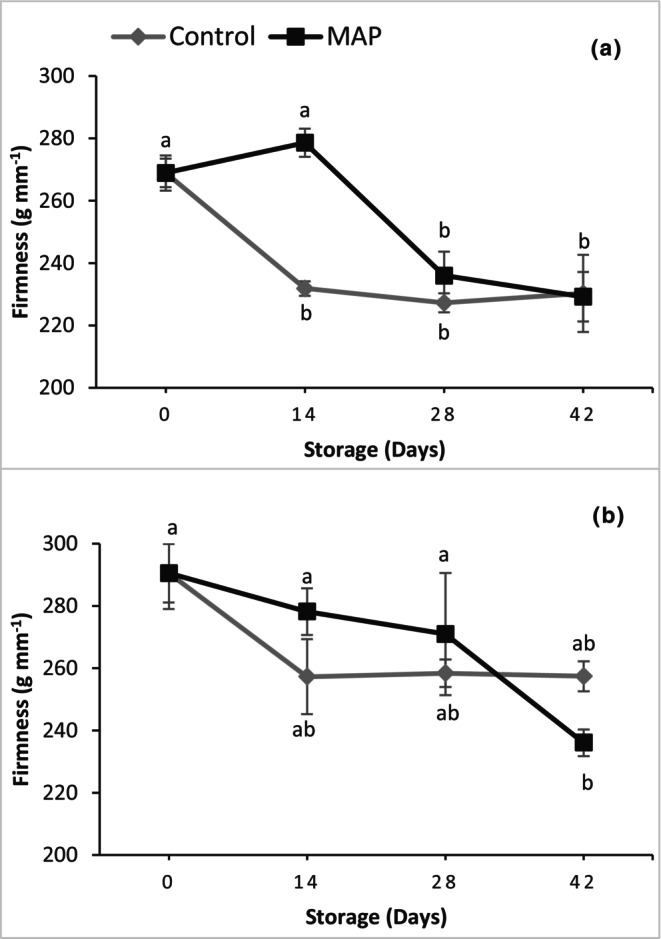
Effect of MAP on berry firmness for (a) 'Granny Val' and (b) 'Supreme' muscadine grape berries over 42‐day storage at 4°C. Data presented here are the mean values and different letters indicate significant differences between treatments and sampling times (*p* ≤ .05).

The firmness of the berries of both grape cultivars declined significantly during storage – by approximately 14.6 and 15.1% for 'Granny Val' and 'Supreme', respectively (Figure 4). Berry firmness of 'Supreme' was not affected significantly by MAP, whereas MAP resulted in significantly higher firmness retention for 'Granny Val' during the early part of the storage duration (Figure 4).

### 3.3 Biochemical attributes

**FIGURE 5:**
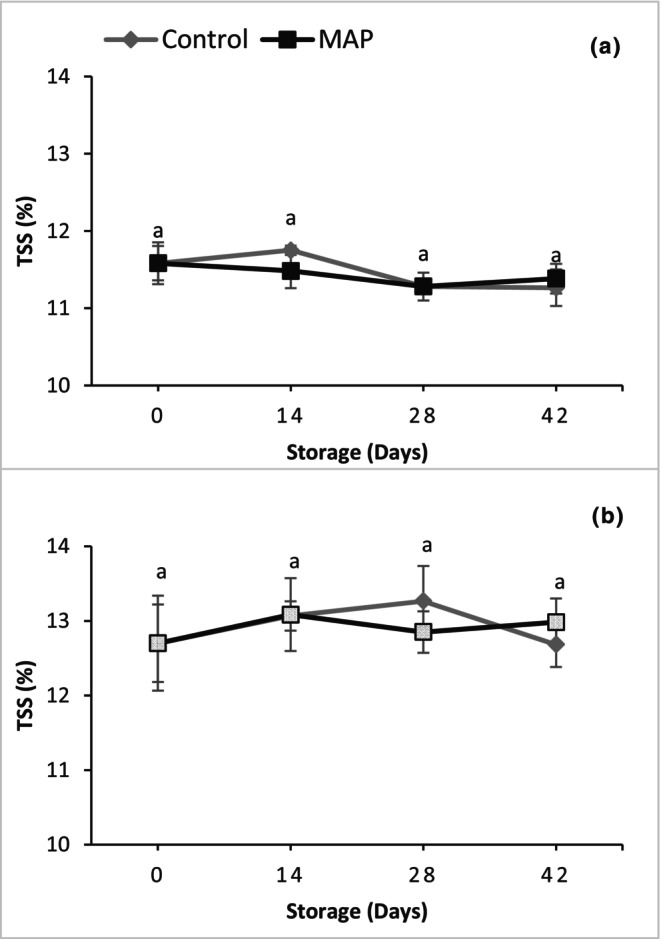
Effect of MAP on total soluble solids for (a) 'Granny Val' and (b) 'Supreme' muscadine grape berries over 42‐day storage at 4°C. Data presented here are the mean values and different letters indicate significant differences between treatments and sampling times (*p* ≤ .05).

**FIGURE 6:**
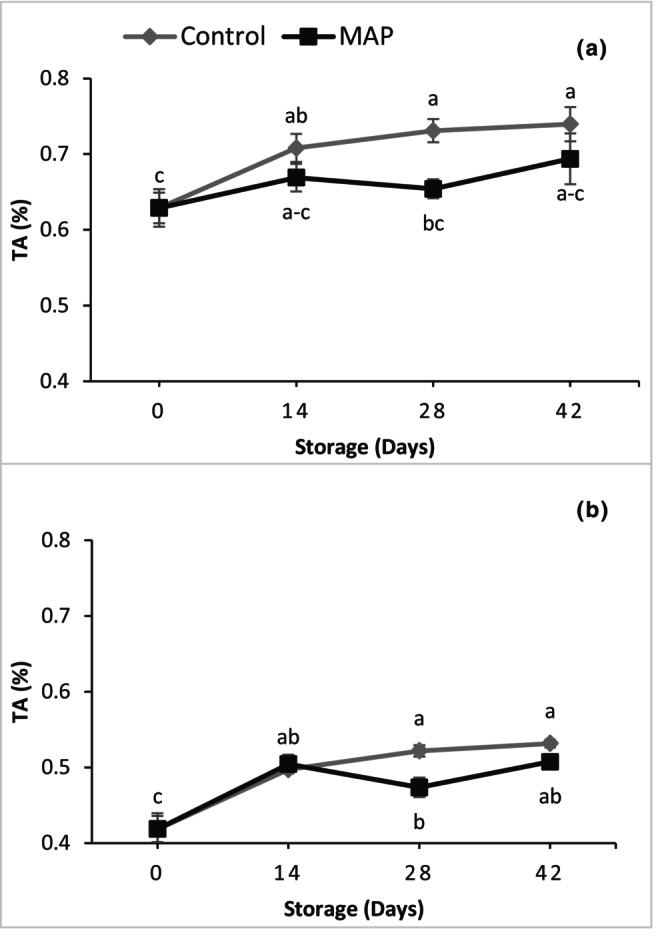
Effect of MAP on titratable acidity for (a) 'Granny Val' and (b) 'Supreme' muscadine grape berries over 42‐day storage at 4°C. Data presented here are the mean values and different letters indicate significant differences between treatments and sampling times (*p* ≤ .05).

There were no significant differences among treatments and storage days for the TSS of both muscadine grape cultivars (Figure 5), whereas TA increased during storage in both cultivars (Figure 6). Regardless of treatment, the TA values for 'Granny Val' and 'Supreme' were 1.14‐ and 1.24‐ fold higher, respectively, at 42 days than on Day 0. The MAP did not affect the TA of 'Supreme' while in 'Granny Val', the TA of the MAP berries remained lower (i.e., closer to the initial value) than the control.

### 3.4 Total antioxidants, total phenolic contents, and anthocyanins

**FIGURE 7:**
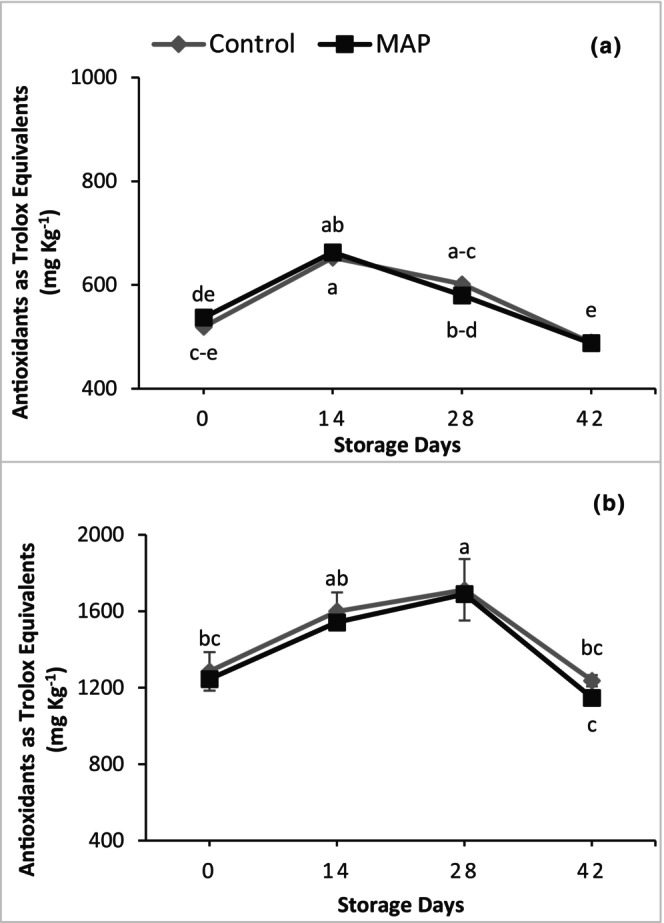
Effect of MAP on total antioxidants for (a) 'Granny Val' and (b) 'Supreme' muscadine grape berries over 42‐day storage at 4°C. Data presented here are the mean values and different letters indicate significant differences between treatments and sampling times (*p* ≤ .05).

Total antioxidants in both cultivars were not affected significantly by the packaging treatments, but there were highly significant changes during storage (Figure 7). Both cultivars showed an increasing trend in total antioxidants initially, which decreased later during storage. In 'Granny Val', total antioxidants increased up to 14 days and afterward decreased linearly until the last day of storage. In 'Supreme', a linear increase in total antioxidants was recorded up to 28 days that decreased later until 42 days. Averaged over the treatments, the maximum antioxidant values for 'Granny Val' and 'Supreme' were 1.25‐fold higher on Day 14 and 1.34‐fold higher on Day 28, respectively, than on Day 0.

**FIGURE 8:**
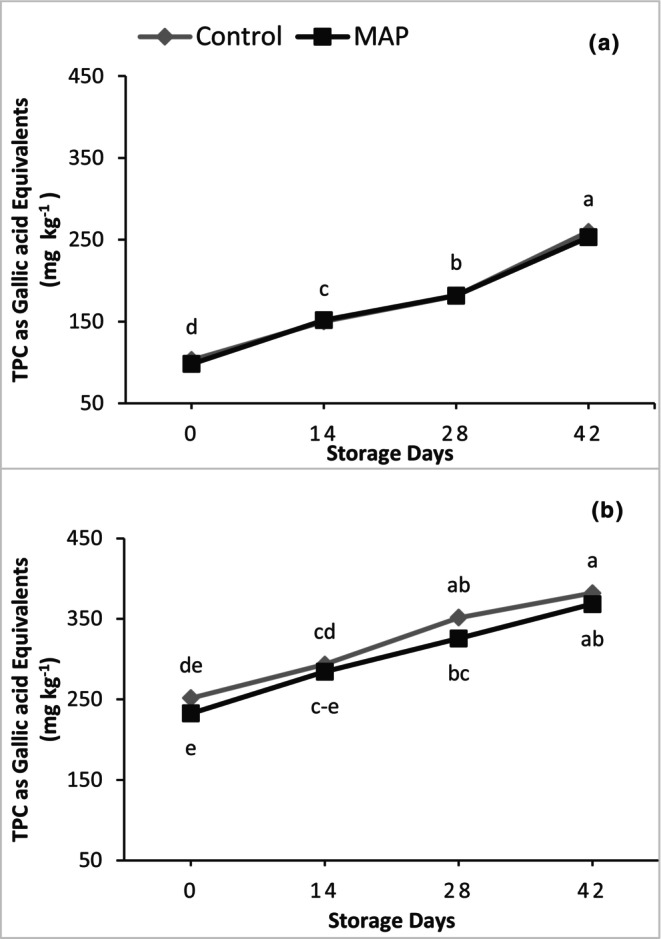
Effect of MAP on total phenolic content (TPC) for (a) 'Granny Val' and (b) 'Supreme' muscadine grape berries over 42‐day storage at 4°C. Data presented here are the mean values and different letters indicate significant differences between treatments and sampling times (*p* ≤ .05).

Like antioxidants, TPC was unaffected by MAP; however, there were significant linear increases in TPC during storage for both grape cultivars (Figure 8). Regardless of MAP treatment, the maximum TPC values were found on the last day of storage when they were 2.55‐fold and 1.55‐fold higher than on Day 0 for 'Granny Val' and 'Supreme', respectively.

**FIGURE 9:**
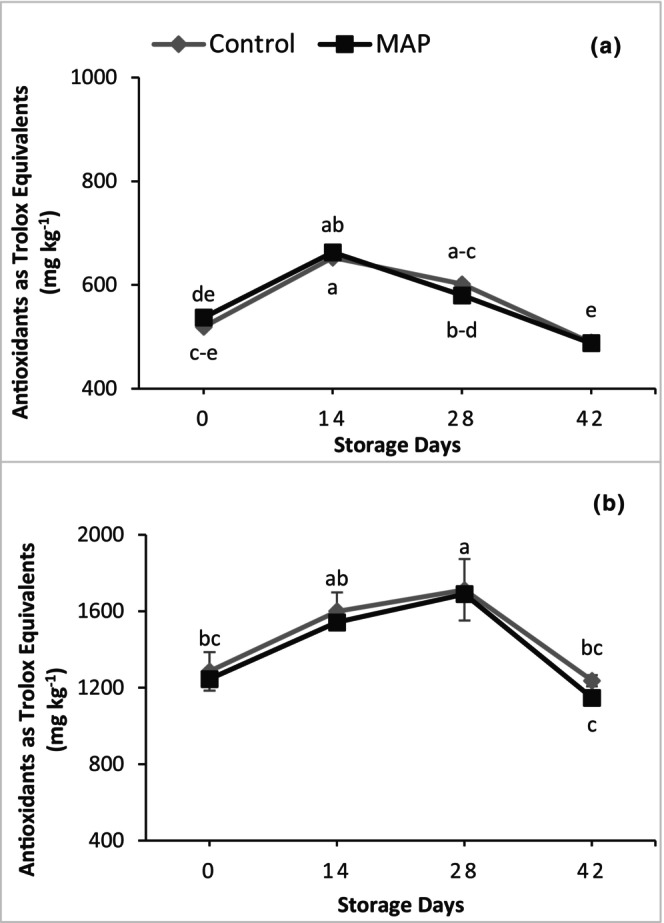
Effect of MAP on total anthocyanins for (a) 'Granny Val' and (b) 'Supreme' muscadine grape berries over a 42‐day storage at 4°C. Data presented here are the mean values and different letters indicate significant differences between treatments and sampling times (*p* ≤ .05).

There were no significant differences in the anthocyanin contents of 'Granny Val' or 'Supreme' grapes in response to MAP treatments; however, the amounts of anthocyanins differed hugely between the two cultivars (Figure 9). For bronze 'Granny Val' berries, the anthocyanin content during storage varied between about 1.2 and 0.4 mg kg^−1^ while for black 'Supreme', the anthocyanin content increased significantly from about 500 mg kg^−1^ on Day 0 to about 800–900 mg kg^−1^ on Day 28, with no further change occurring afterward.

## 4 DISCUSSION

Muscadine grapes, being a delicate fruit, cannot be stored for more than about 2 weeks without postharvest interventions. Refrigerated storage in MAP has been proven effective in maintaining better shelf life of numerous perishable commodities. Moreover, higher CO_2_ and lower O_2_ have been reported to increase the storage life of muscadine, primarily due to the CO_2_ benefits (Shahkoomahally et al., 2021). In the current study, a MAP that was developed through a patented membrane technology (BreatheWay®, Curation Foods, USA) can adapt to temperature fluctuations and respiration changes. MAP was tested in cold storage to determine the impact on shelf life, quality, and composition of two muscadine grape cultivars. In our study, the CO_2_ concentration in MAP was increased from almost 0% to about 4%–6% and the O_2_ was decreased from 20.8% to about 14%–15%. These gas conditions proved to be beneficial environment for muscadine grape storage, particularly in reducing fungal decay.

It was found that MAP can reduce decay incidence significantly without having any impact on berry weight loss. The elevated concentration of CO_2_ maintained by MAP in the current work was previously shown to help in reducing disease incidence of muscadine during CA storage (Shahkoomahally et al., 2021). Exposure to elevated CO_2_ has also been reported to reduce decay incidence of common table grapes in some other studies (Candir et al., 2012; Romero et al., 2019).

Weight loss was not significantly different in the control and MAP treatments as berries in both treatments were stored in sealed trays under cold storage. Similar results of insignificant weight loss have also been reported in some other studies of packaged table grapes (Martínez‐Romero et al., 2003; Valverde et al., 2005). Among various color parameters, L* and *Hue* are important to describe changes in postharvest fruit quality (Valverde et al., 2005). In the current experiment, L* and *Hue* decreased linearly during storage for 'Granny Val' as the skin darkened slightly (Figures S2 and S3), but MAP resulted in better retention of L* during storage, which agrees with the results of Shahkoomahally et al. (2021). Changing of the color from bright to dull could be due to changes in the TPC (Himelrick, 2003), which increased during storage. Berry firmness of 'Granny Val' was better maintained in MAP for 14 days at 4°C, but was otherwise not affected significantly by MAP in either cultivar as the firmness declined over the entirety of storage, which could be related to the decrease in total pectin during storage of muscadines as previously reported (Himelrick, 2003).

The TSS and TA are important parameters to determine the organoleptic quality of dessert fruit like muscadine. In the current study, TSS was not affected by MAP treatment and did not change significantly during storage, but TA was maintained slightly lower in MAP as it increased during storage. No differences in TSS during storage have also been reported in different studies on both table grapes and muscadine (Barchenger et al., 2015; Romero et al., 2019). Marsh et al. (2004) reported an increase in malate content during storage of kiwifruit at 4°C as compared to 0°C. Thus, storage temperature can affect the ratio of the major acids in fruit. This could be a possible reason for the increase in acidity of muscadine grape berries stored at 4°C in the current study.

Fruit suffer from stress during storage as nutrient and water supply from the parent plant is discontinued, which results in activating defense mechanisms. Different enzymatic and nonenzymatic antioxidants are produced under such postharvest storage stress (Xu et al., 2009). Phenylalanine ammonia‐lyase (PAL) is among the enzymes whose activity can increase in response to stress, resulting in increasing TPC (Nia et al., 2021; Shah et al., 2022). In the current study, an increase in TPC and antioxidants during storage could be due to the defense mechanism of the fruit against postharvest storage stress. Moreover, TPC during storage could also increase due to enhanced supply of carbon skeleton and degradation of organic acids (Maurer et al., 2017). After a certain period of storage, the decrease in antioxidants observed here might be due to fruit aging and senescence, which had previously been observed in grapes (Khalil, Rajwana, Razzaq, Farooq, et al., 2023; Nia et al., 2021).

Anthocyanins are pigments and their virtual absence in 'Granny Val' is not surprising being a bronze cultivar. 'Supreme', a black muscadine cultivar, contained higher anthocyanin content than 'Granny Val', which also increased during storage. A similar increase in some cultivars of muscadine was also reported by Marshall‐Shaw et al. (2014) who, like in the present study, also found a positive correlation between TPC and anthocyanins.

## 5 CONCLUSION

The current study provides evidence of improved quality retention of muscadine grapes during storage by using MAP. Fruit stored in MAP had lower decay with better berry firmness and color retention, especially up to 28 days of storage at 4°C. Even after 42 days of storage, the decay incidence was significantly lower in MAP, whereas the biochemical attributes were not affected. Further studies should be carried out with MAP and muscadine using even higher CO_2_ concentrations than the current experiment as muscadine grapes are highly tolerant of elevated CO_2_ levels.

## AUTHOR CONTRIBUTIONS

**Uzman Khalil:** Formal analysis (lead); investigation (lead); methodology (equal); resources (lead); validation (equal); writing – original draft (lead). **Ishtiaq A. Rajwana:** Conceptualization (equal); project administration (equal); validation (equal). **Kashif Razzaq:** Formal analysis (supporting); methodology (equal); supervision (equal); writing – review and editing (equal). **Shehbaz Singh:** Methodology (equal); resources (lead). **Ali Sarkhosh:** Conceptualization (equal); methodology (equal); supervision (equal); validation (equal); writing – review and editing (equal). **Jeffrey K. Brecht:** Conceptualization (lead); project administration (equal); resources (equal); supervision (lead); writing – review and editing (lead).

## FUNDING INFORMATION

Mr. Uzman Khalil gratefully acknowledges the Higher Education Commission of Pakistan for providing financial support under the International Research Support Initiative Program to perform this research at the University of Florida, United States of America.

## CONFLICT OF INTEREST STATEMENT

The authors declare no conflict of interest to disclose.

## Supporting information


Figure S1.

Figure S2.

Figure S3.

## Data Availability

The data that support the findings of this study are available from the corresponding author upon reasonable request.
