# Supplementary material for: Evaluation of modified atmosphere packaging system developed through breathable technology to extend postharvest life of fresh muscadine berries
Source: Food Sci Nutr. 2024 Mar 18;12(5):3663–73. doi: 10.1002/fsn3.4037 (PMC11077196; doi:10.1002/fsn3.4037)
Supplement: Supplementary file 1 — Figure S1. [file FSN3-12-3663-s003.docx]

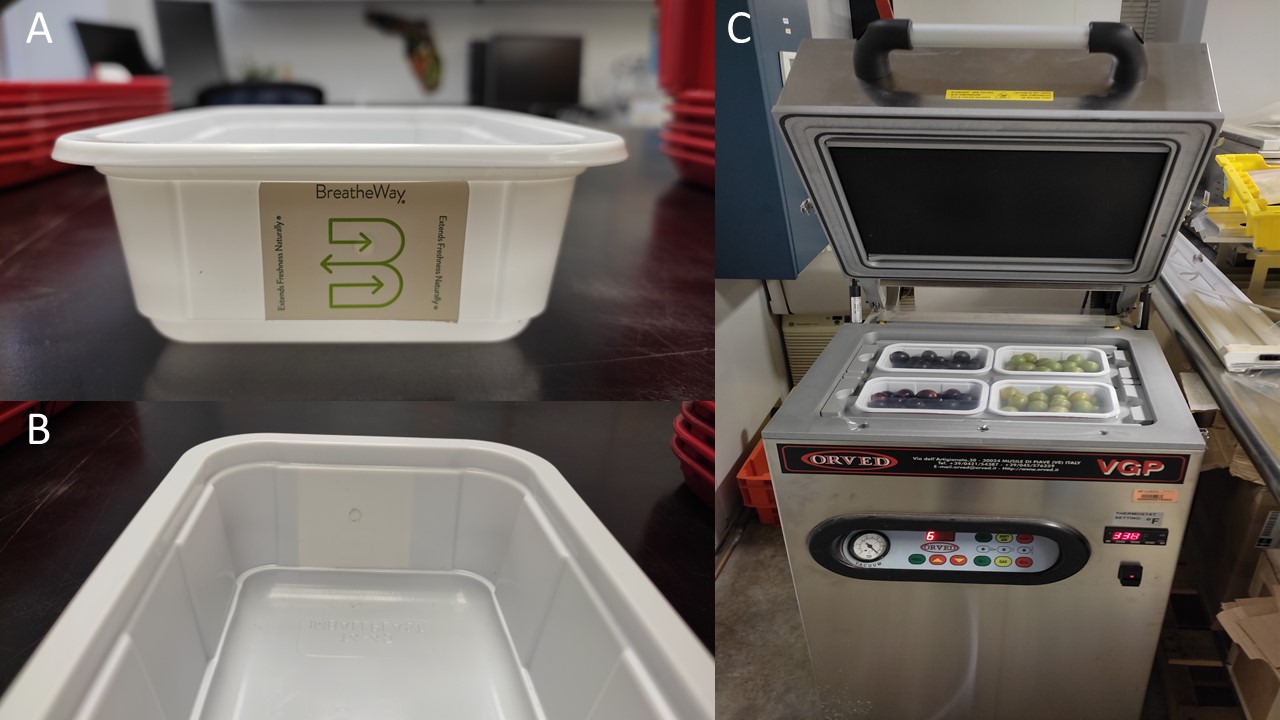

**Supplemental Fig 1.** Pictorial view of packaging used from outside (A), inside (B) and during sealing with impermeable film (C).
