# Supplementary material for: Evaluation of modified atmosphere packaging system developed through breathable technology to extend postharvest life of fresh muscadine berries
Source: Food Sci Nutr. 2024 Mar 18;12(5):3663–73. doi: 10.1002/fsn3.4037 (PMC11077196; doi:10.1002/fsn3.4037)
Supplement: Supplementary file 2 — Figure S2. [file FSN3-12-3663-s002.docx]

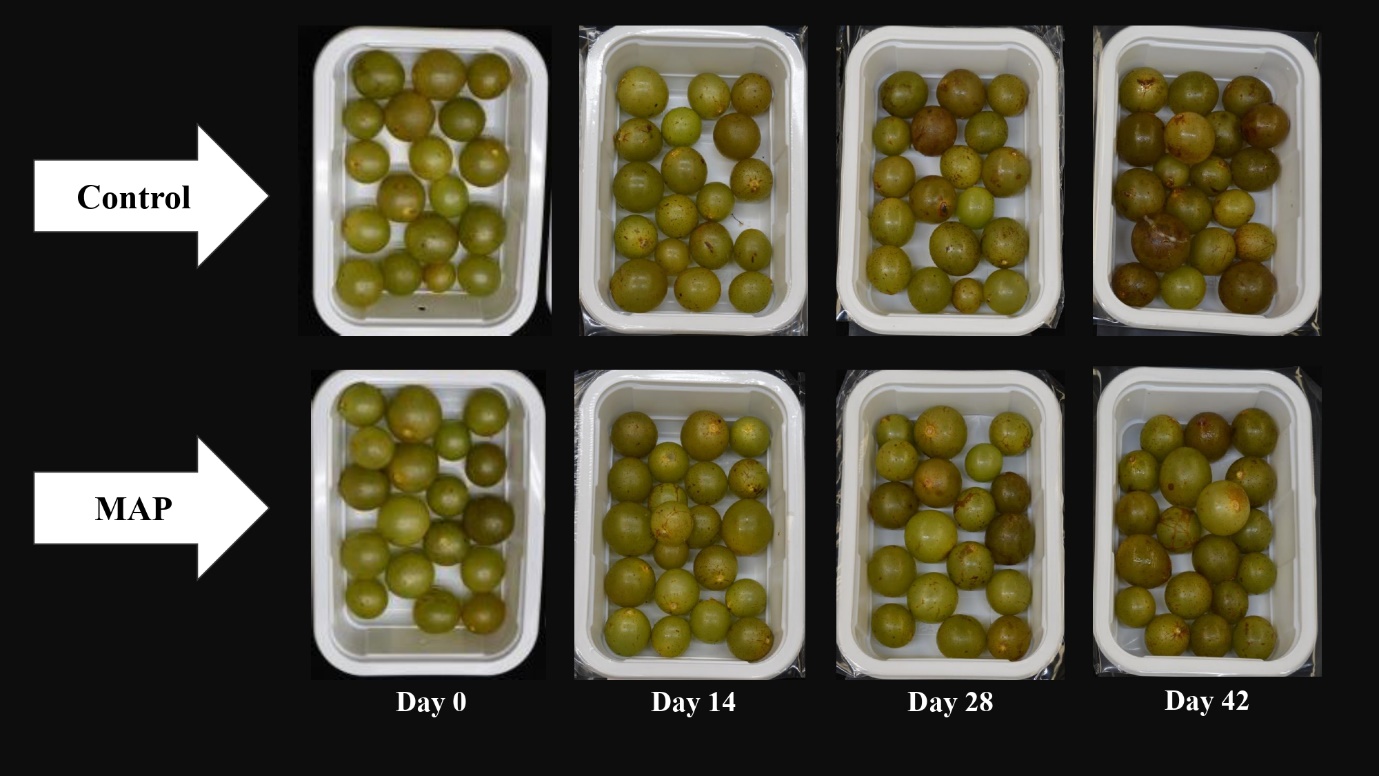

**Supplemental Fig 2.** Pictorial view of ‘Granny Val’ muscadine grape berries packaged in Control and MAP trays for 42 days at 4 ºC.
