# Supplementary material for: Evaluation of modified atmosphere packaging system developed through breathable technology to extend postharvest life of fresh muscadine berries
Source: Food Sci Nutr. 2024 Mar 18;12(5):3663–73. doi: 10.1002/fsn3.4037 (PMC11077196; doi:10.1002/fsn3.4037)
Supplement: Supplementary file 3 — Figure S3. [file FSN3-12-3663-s001.docx]

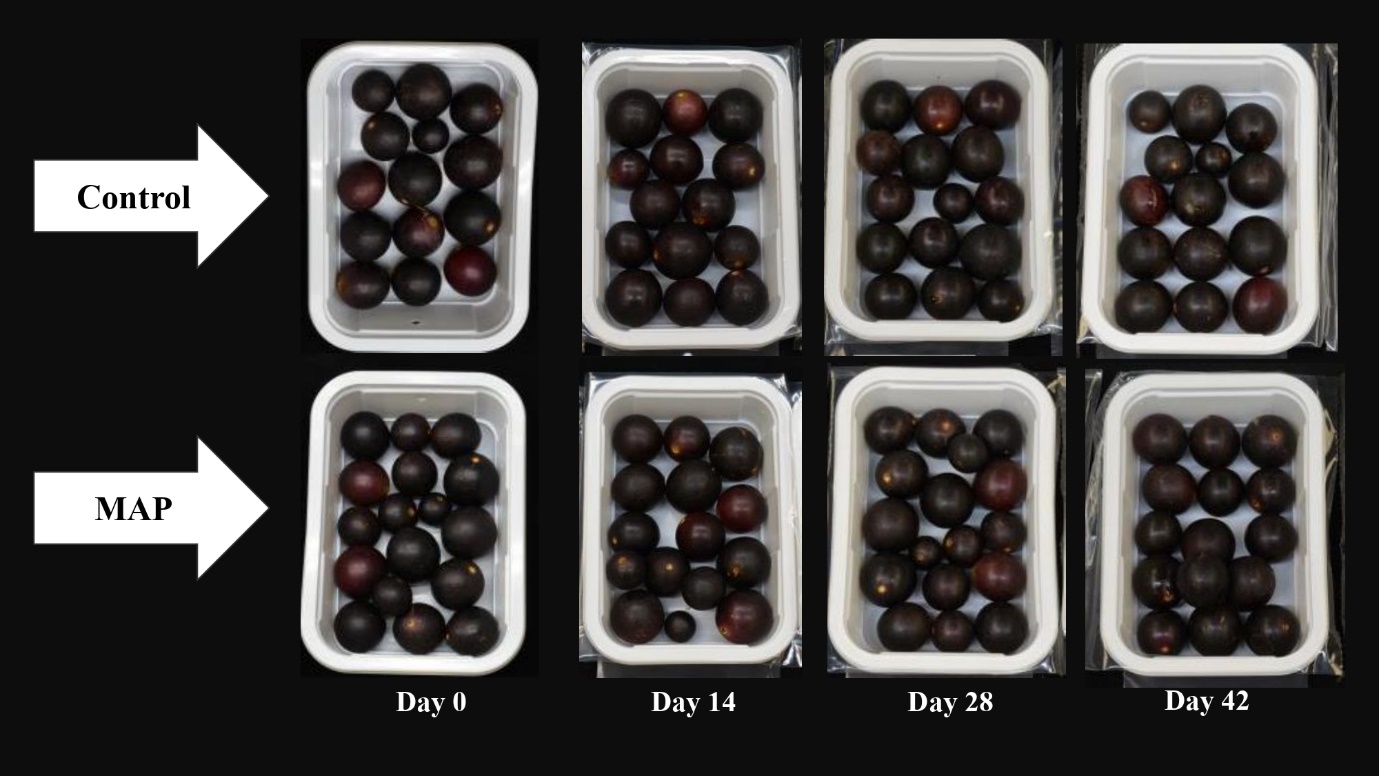

**Supplemental Fig 3.** Pictorial view of ‘Supreme’ muscadine grape berries packaged in Control and MAP trays for 42 days at 4 ºC.
